# Operational research: did health systems in Sierra Leone recover after the 2014-15 Ebola outbreak?

**DOI:** 10.12688/f1000research.19077.1

**Published:** 2019-06-06

**Authors:** Tom Decroo, Alexandre Delamou, John C. Reeder

**Affiliations:** 1 Department of Clinical Sciences, Institute of Tropical Medicine, Antwerp, Antwerp, 2000, Belgium; 2 Department of Public Health, Gamal Abdel Nasser University, Conakry, Guinea; 3 Special Programme for Research and Training in Tropical Diseases, UNICEF/UNDP/World Bank/WHO, Geneva, Switzerland

**Keywords:** Ebola Virus Disease, emergency, operational research, implementation research, outbreak response

## Abstract

Findings from participants who attended the second post-Ebola SORT-IT course are reported in five short papers on the “legacy of Ebola on health Systems in Sierra-Leone”. After a decline of health service provision and utilisation during the Ebola outbreak, present findings show recovery over time in the post-Ebola period. However, important challenges remain, including an important health workforce gap.

## Operational research studies on the 2015–2015 Ebola outbreak

Operational research studies, using the Structured Operational Research Initiative (SORT-IT) model ^1^, assessed the effect of the devastating 2014–2015 Ebola outbreak on West-African health systems during and immediately after the outbreak ^2^. Findings from participants who attended the second post-Ebola SORT-IT course are reported here in five short papers on the “legacy of Ebola on health Systems in Sierra-Leone”. Consideration of these findings is an important step in building resilience to future challenges.

After a decline of health service provision and utilisation during the outbreak ^2, 3^, present findings show recovery over time in the post-Ebola period. There are some encouraging signs that some health indicators may even be better than before the outbreak. Following investments in staff and community involvement, a steep increase of utilization of malaria diagnostic testing was reported post-Ebola ^3^. In addition, post-Ebola childhood mortality is reported to be lower compared to pre-Ebola ^4^. Encouragingly, the Community Health Worker programme, with a focus on the diagnosis and treatment of malaria, pneumonia and diarrhea, performed consistently throughout the pre-, intra-, and post-Ebola period ^5^.

However, some important challenges still remain. Although service utilization has increased, staff shortages remain unaddressed. Medical staff and non-medical staff deficits hover around 60% and 90%, respectively, and did not substantially change post-Ebola ^6^. The recovering health system was unable to cope with the increased demand for anti-malaria drugs, which resulted in stock outs ^3^. In addition, a 12-fold increase of measles cases post-Ebola is an alarming indicator of reduced herd immunity and requires urgent action ^4^. Finally, care for non-communicable diseases (NCD) has not yet returned to pre-Ebola levels ^7^.

To fully recover from the Ebola outbreak, the health systems, including decentralized health care provision, needs to be strengthened further. Training of health care workers, robust information systems and reliable drug supply are needed to further increase and maintain access to health care provision. Moreover, community involvement is needed to re-establish trust, promote long-term utilization of health services and support disease surveillance and eventual future outbreak response ^8, 9^. From the 2014–15 Ebola outbreak operational research initiatives we learnt that capacity building in programme data collection, analysis and reporting and timely access to information are absolutely needed to ensure learning-by-doing and evidence-informed adaptations to practice and policies during outbreak response ^10^. Open access publication of operational research manuscripts even before the peer review process is completed, for instance through the F1000 portal, is a mean to achieve this. Moreover, in the post-Ebola era, more operational research such as that reported here will be key to inform programmes about progress beyond the pre-Ebola health status, and to monitor the journey to the Sustainable Development Goal “ensure healthy lives and promote wellbeing for all at all ages”.

## Data availability

No data is associated with this article
